# Effects of molecular hydrogen supplementation on fatigue and aerobic capacity in healthy adults: A systematic review and meta-analysis

**DOI:** 10.3389/fnut.2023.1094767

**Published:** 2023-02-02

**Authors:** Kaixiang Zhou, Meng Liu, Yubo Wang, Haoyang Liu, Brad Manor, Dapeng Bao, Luyu Zhang, Junhong Zhou

**Affiliations:** ^1^College of Sports and Health, Chengdu University of Traditional Chinese Medicine, Chengdu, Sichuan, China; ^2^Sports Coaching College, Beijing Sport University, Beijing, China; ^3^China Institute of Sport and Health Science, Beijing Sport University, Beijing, China; ^4^Hebrew SeniorLife Hinda and Arthur Marcus Institute for Aging Research, Harvard Medical School, Boston, MA, United States; ^5^School of Strength and Conditioning Training, Beijing Sport University, Beijing, China

**Keywords:** molecular hydrogen, fatigue, aerobic capacity, rating of perceived exertion, maximal oxygen uptake

## Abstract

**Background:**

Fatigue is oftentimes induced by high-intensity exercise potentially *via* the exceeded amount of reactive oxygen species, leading to diminished functions (e.g., aerobic capacity) and increased risk of injuries. Studies indicate that molecular hydrogen (H_2_), with antioxidant and anti-inflammatory properties, may be a promising strategy to alleviate fatigue and improve aerobic capacity. However, such effects have not been comprehensively characterized.

**Objective:**

To systematically assess the effects of in taking H_2_ on fatigue and aerobic capacity in healthy adults.

**Methods:**

The search was conducted in August 2022 in five databases. Studies with randomized controlled or crossover designs that investigated the rating of perceived exertion (RPE), maximal oxygen uptake (VO_2max_), peak oxygen uptake (VO_2peak_), and endurance performance were selected. The data (mean ± standard deviation and sample size) were extracted from the included studies and were converted into the standardized mean difference (SMD). Random-effects meta-analyses were performed. Subgroup analysis was used to analyze potential sources of heterogeneity due to intervention period, training status, and type of exercise.

**Results:**

Seventeen publications (19 studies) consisting of 402 participants were included. The pooled effect sizes of H_2_ on RPE (SMD_pooled_ = −0.38, 95%CI −0.65 to −0.11, *p* = 0.006, *I*^2^ = 33.6%, *p* = 0.149) and blood lactate (SMD_pooled_ = −0.42, 95% CI −0.72 to −0.12, *p* = 0.006, *I*^2^ = 35.6%, *p* = 0.114) were small yet significant with low heterogeneity. The pooled effect sizes of H_2_ on VO_2max_ and VO_2peak_ (SMD_pooled_ = 0.09, 95% CI −0.10 to 0.29, *p* = 0.333, *I*^2^ = 0%, *p* = 0.998) and endurance performance (SMD_pooled_ = 0.01, 95% CI −0.23 to 0.25, *p* = 0.946, *I*^2^ = 0%, *p* > 0.999) were not significant and trivial without heterogeneity. Subgroup analysis revealed that the effects of H_2_ on fatigue were impacted significantly by the training status (i.e., untrained and trained), period of H_2_ implementation, and exercise types (i.e., continuous and intermittent exercises).

**Conclusions:**

This meta-analysis provides moderate evidence that H_2_ supplementation alleviates fatigue but does not enhance aerobic capacity in healthy adults.

**Systematic review registration:**

www.crd.york.ac.uk/PROSPERO/, identifier: CRD42022351559.

## 1. Introduction

Aerobic capacity enables the performance of daily activities that require repetitive movements of the body for prolonged periods of time and/or against physical loads (e.g., exercise) (1). Fatigue induced by such activities is a significant contributor to reduced performance, as well as to exhaustion and weakness (2–5). Considerable effort has therefore been taken to develop safe strategies to effectively reduce fatigue and in turn improve aerobic capacity within both the sport and non-sport setting.

One mechanism of fatigue development appears to be that high-intensity exercise induces high amounts of reactive oxygen species (ROS) within mitochondria, which leads to dysregulation within human inflammatory and neuroendocrinological systems (6–10). Studies have thus emerged to implement antioxidant nutrients, such as vitamins and resveratrol, to alleviate fatigue and facilitate recovery from fatigue (11, 12). These studies have shown promise, but have also highlighted that the appropriate dosage of these nutrients is critical and that the intake of excessive amounts may induce side effects such as oxidative stress and the inhibition of exercise-induced physiological adaptations within skeletal muscle and the cardiovascular system (12–16).

Since Ohsawa's pioneering discovery of the selective antioxidant function of molecular hydrogen (H_2_), research suggests that the intake of H_2_ holds promise as a non-toxic strategy to alleviate exercise-induced oxidative stress and inflammation (17–19). Studies have reported that H_2_ molecules administered *via* inhaled gas or oral water, can penetrate cell membranes and diffuse rapidly into organelles (20), thus selectively reducing OH and ONOO^−^ (21, 22). Recently, a number of relatively small studies have also demonstrated that the intake of H_2_ *via* hydrogen-rich water (HRW) or hydrogen-rich gas (HRG) may reduce fatigue and enhance aerobic capacity (23–27). However, results of these studies have been inconsistent. For example, one study observed that intake of 500 mL hydrogen-rich water within 10 min before exercise increased maximum oxygen uptake (VO_2max_) and reduced subjective fatigue during an incremental cycling exercise in healthy young adults (25). In contrast, another study reported that intake two doses of 290 mL hydrogen-rich water before an incremental treadmill running exercise could not induce such benefits in endurance athletes (28). These and other studies suggest that the impact of H_2_ supplementation may depend upon dosage, as well as other factors including training status of the individual and the type of exercise in question(23, 29, 30).

The purpose of this study was to examine the impact of H_2_ intake on fatigue and aerobic capacity by conducting a systematic review and meta-analysis of available peer-reviewed publications on the topic. Subgroup analyses were also completed in hopes of guiding future research by providing insight into whether dosage, training status, and exercise types potentially influences the impact of H_2_ supplementation. Our overarching hypothesis was that H_2_ supplementation prior to or during exercise would significantly reduce fatigue and increase aerobic capacity in healthy adults.

## 2. Methods

This study was conducted in accordance with the Preferred Reporting Items for Systematic Reviews and Meta-analyses (PRISMA) guidelines (31). This study was registered with PROSPERO (CRD42022351559).

### 2.1. Data sources, searches, and study selection

Two authors (KZ and ML) independently searched PubMed, Web of Science, Medline, Sport-Discus, and PsycINFO databases from inception to August 5, 2022, using a comprehensive search strategy (eTable 1). Manual searches of the reference lists in the related publications were also performed.

Studies were included if: (1) the participants were healthy adults with a mean age≥18 years and were free from any dietary supplements or medications; (2) the intervention was the intake of molecular hydrogen by the participants; (3) the control group with placebo; (4) the outcomes include at least one of RPE, blood lactate, VO_2max_, peak oxygen uptake (VO_2peak_), and performance of endurance exercises (i.e., cycling time to exhaustion, race time, etc.); (5) randomized controlled or crossover design.

Studies were excluded if they were: (1) animal trials; (2) written by non-English language, (3) without specific data; (4) review and conference articles; and (4) repeated publications.

### 2.2. Data extraction, outcomes, and risk of bias assessment

Two independent reviewers (ML and YW) extracted relevant data from each included study (32), including the authors, publication year, sample size, participant characteristics, H_2_ administration protocol, design of exercise, and outcome measures. Any disagreement between the two authors was discussed with JZ and DB until a consensus was achieved.

The primary outcome of fatigue was the RPE score and that of aerobic capacity was VO_2max_, or VO_2peak_ (33, 34) when VO_2max_ was not available. The secondary outcome of fatigue was blood lactate and that of aerobic capacity was performance of endurance exercises, including cycling time to exhaustion, and race time. The mean and standard deviation of each outcome in post-tests in each study were extracted. The post-test data of outcomes in each study were summarized in eTable 2.

Two investigators (KZ and ML) independently assessed the risk of bias in the included studies using the Cochrane Collaboration's tool (35), containing the following criteria: (1) selection bias; (2) performance bias; (3) detection bias; (4) attrition bias; (5) reporting bias; (6) other sources of bias. Studies were defined as high risk of bias when ≥1 of these items were with a high risk of bias, and as low risk if all these items were with low risk of bias. In other situations, it was defined as moderate risk.

### 2.3. Statistical analysis and grading the evidence

To determine the effect size (ES) of the intervention, the standardized mean difference (SMD; Hedges' g) of the outcomes was calculated, with a 95% confidence interval (CI). ES was classified as trivial (<0.2), small (0.2~0.49), moderate (0.5~0.79), or large (>0.8) (36). Meta-analysis was performed in Stata v15.1 (STATA Corp., College Station, TX) using the inverse variance method. Heterogeneity was assessed by measuring the inconsistency (*I*^2^ statistic) of intervention effects among the trials. The level of heterogeneity was interpreted according to guidelines from the Cochrane Collaboration: trivial (<25%), low (25~50%), moderate (50~75%), and high (>75%) (37). A random-effects model was used to estimate the pooled effect in anticipation of heterogeneity across the studies due to differences in participants and intervention characteristics. The publication bias was assessed by the funnel plot and Egger's test. Subgroup analysis was used to analyze potential sources of heterogeneity due to intervention period, training status, and type of exercise. If a significant asymmetry was detected, we used the Trim and Fill method for sensitivity analysis of the results (38). All the statistical significance was set at *p* < 0.05.

Additionally, the quality of evidence for outcomes was evaluated using the Grading of Recommendations Assessment, Development and Evaluation (GRADE), which characterizes the evidence on the study limitations, imprecision, inconsistency, indirectness, and publication bias (39, 40).

## 3. Results

**Figure 1:**
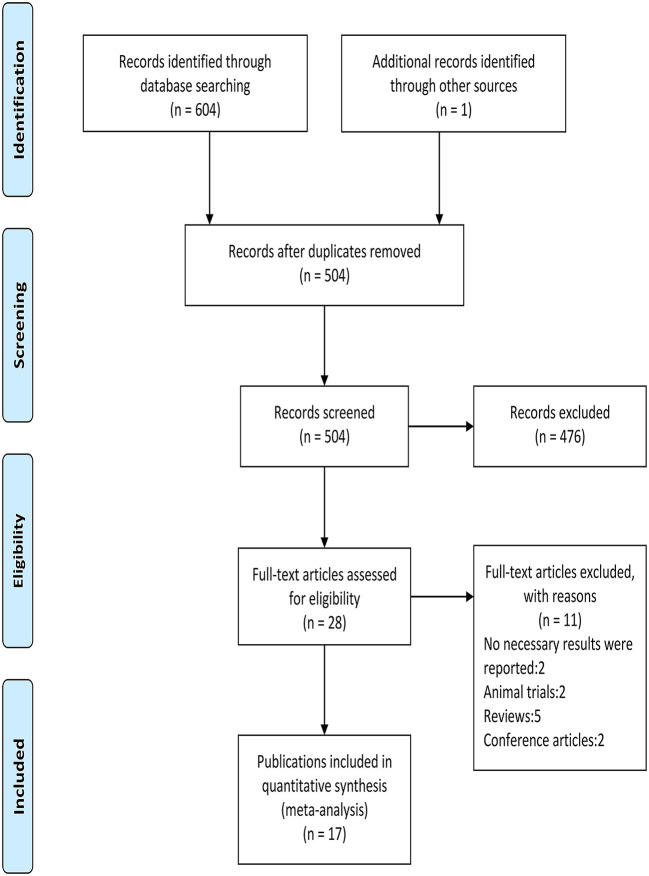
Study flowchart.

The flow diagram of screening is shown in Figure 1. A total of 605 relevant publications were retrieved (PubMed *n* = 50, Web of Science *n* = 305, Medline *n* = 210, Sport-Discus *n* = 36, PsycINFO *n* = 3, Manual search *n* = 1), and 577 publications were excluded after reviewing the titles and abstracts. After the evaluation of full texts, 11 of the 28 publications were removed, and thus 17 publications consisting of 19 studies (i.e., 15 randomized crossover designs and four randomized controlled trials) were included in the following analyses (Table 1). One publication (25) included two randomized controlled trials, and the other one (23) included a randomized crossover study and a randomized controlled trial.

**Table 1:** Characteristics of the included studies (*n* = 19).

**Study**	**Design**	**Total no. of participants**	**Age, mean ±SD, y**	**No. of participants by sex (%)**	**Training status**	**Protocol of H_2_ administration**	**Exercise protocol**	**Outcome measures**
Aoki et al. (42)	RCD	10	20.9 ± 1.3	Male:10(100) Female:0(0)	Elite socc er players	HRW (H_2_ conc.:0.92~1.02 ppm) Three 500 ml doses before exercise	Cycling for 30 min at 75 % VO_2max_ and maximal knee extension exercise	Fatigue: BLA↓ Others: d-ROMs → ; BAP → ; CK → ; Peak torque → ; MF↓; MPF↓
Drid et al. (43)	RCD	8	21.4 ± 2.2	Male:0(0) Female:8(100)	Judo athletes	HRW 300 mL within 30 min before exercise	Special judo fitness test	Fatigue: BLA↓ Aerobic capacity: Performance index → Others: pH↓;Bicarbonate↓;HR_max_ → ;Resting HR → ; Recovery HR →
Da Ponte et al. (44)	RCD	8	41 ± 7	Male:8(100) Female:0(0)	Well-trained cyclists	HRW (pH:9.8; ORP:−180 mV; FH:450 ppb; TDS:180 mg/L) 2 liters per day for 2 weeks before exercise	30 min intermittent cycling test to exhaustion	Fatigue: RPE^c^ → ; BLA → Aerobic capacity: P_m_→ Others: VO_2_ → ; RER → ; HR_avg_ → ; HR_max_ → ; P_max_ → ; Fatigue index → ; Time to P_max_ → ; Blood pH → ; Bicarbonate [HCO${}_{3}^{-}]\to$; Base excess → ; pO_2_ → ; pCO_2_ → ; Hemoglobin → ; Hemoglobin Sat → ; Glucose →
LeBaron et al. (45)	RCD	19	25.0 ±8.9	Male:15(79) Female:4(21)	Untrained healthy participants	HRW (TDS:13.1 mg/L)	Incremental treadmill running test to exhaustion	Aerobic capacity: VO_2peak_→ Others: HR_avg_↓; HR_max_ → ; RER → ; RR →
						500 ml intake the day before and on the day of exercise		
Botek et al. (26)	RCD	12	27.1 ± 4.9	Male:12(100) Female:0(0)	Recreationally trained sport science students	HRW (pH:7.4; ORP:−400 mV; Temp: 22°C; H_2_ conc.:0.5 ppm)	Incremental cycling test to exhaustion	Fatigue: RPE↓; BLA↓ Others: VE → ; VO_2_ → ; VE/VO_2_↑; HR_avg_ → ; RQ →
						600 ml within 30 min before exercise		
Javorac et al. (27)	RCD	20	22.9 ± 1.5	Male:10(50) Female:10(50)	Untrained physically active participants	HRG (%4 H_2_) 20 min once-per-day inhalation for 7 days	Incremental treadmill running test to exhaustion	Fatigue: BLA → Aerobic capacity: VO_2max_ → ; TTE → Others: Leg MVIS → ;YMCA endurance → ; resting Blood pressure → ; Resting HR → ; MRS↑; Insulin → ; Ghrelin → ; IGF-1↑; CK → ; Myoglobin → ; C-reactive protein↑; Ferritin↑; ESR →
Ooi et al. (28)	RCD	14	34 ± 4	Male:14(100) Female:0(0)	Well-trained runners/triathletes	HRW (H_2_ conc.: 2.60 ppm) 2 doses of 290 mL within 5~10 min before exercise	Incremental treadmill running test to exhaustion	Fatigue: RPE → ; BLA → Aerobic capacity: VO_2max_ → ; TTE → Others: RE → ; Speed at OBLA → ; HRmax → ; VE_max_ → ; RER → ; Blood Glucose → ; Blood HCO${}_{3}^{-}\to$; Blood pH →
Mikami et al. (25)^a^	RCT	H:52	51.2 ± 6.9	Male:23(44) Female:29(56)	Untrained physically active participants	HRW (H_2_ conc.:0.8 ppm) 500 mL within 30 min before exercise	Incremental cycling test to 75% HRmax	Fatigue: RPE↓ Aerobic Capacity:VO_2max_→ Others: Resting HR↓; VAS↓
		P:47	51.5 ± 7.9	Male:20(43) Female:27(57)				
Mikami et al. (25)^b^	RCT	H:30	43.6 ± 13.3	Male:15(50) Female:15(50)	Fitness trainers	HRW (H_2_ conc.:1.0 ppm) 500 mL within 10 min before exercise	Incremental cycling test to HRmax	Fatigue: RPE↓ Aerobic capacity: VO_2max_↑
		P:30	43.2 ± 14.4	Male:15(50) Female:15(50)				
Dobashi et al. (47)	RCD	8	19.4 ± 0.85	Male:8(100) Female:0(0)	Untrained physically active participants	HRW (Temp:4°C; H_2_ conc.:5.14 ppm)	6 min repeated sprint cycling exercise	Fatigue: BLA → Others: CMJ → ; MVIC → ; P_max_; P_m_ for 10-s → ; d-ROMs → ; BAP →
						500 mL within 5 min before and after the exercise for 3 days		
Botek et al. (30)	RCD	16	31.6 ± 8.6	Male:16(100) Female:0(0)	Well-trained runners	HRW (pH:7.8; H_2_ conc.: 0.9 ppm) 420-mL doses at 24 h, 3 h, 2 h, and 40 min before exercise	4.2-km up-hill race	Fatigue: RPE → Aerobic capacity: Race time → Others: HR_max_→
Shibayama et al. (48)	RCD	8	20.9 ± 0.3	Male:8(100) Female:0(0)	Untrained physically active participants	HRG (68% H_2_) 60 min after exercise	30min Treadmill running (75%VO_2max_) and squat jump 5 × 10 rep.	Aerobic capacity: P_m_→ Others: CMJ↑; MVIC → ; P_max_ → ; d-ROMs → ; BAP → ; U8ER↓; CKa → ; LDa → ; White blood cells →
Hori et al. (29)	RCD	12	21.8 ± 5.8	Male:12(100) Female:0(0)	Untrained healthy participants	HRG (1% H_2_) 30 min during exercise	Cycling for 30 minutes at 60% VO_2peak_	Aerobic capacity: VO_2peak_→ Others: VCO_2_↑; VE↑; HRavg → ; Vacetone↑; VO_2_ rest → ; VCO_2_ rest → ; VE rest → ; Recovery HR → ; Vacetone rest → ; d-ROMs → ; BAP →
Hori et al. (23)^a^	RCD	9	19.9 ± 1.2	Male:6(67) Female:3(33)	Untrained university students	HRW (H_2_ conc.:4.3 ppm)	Incremental cycling test to exhaustion	Fatigue: RPE → ; BLA → Aerobic capacity: VO_2peak_→ Others: Resting HR → ; P_max_ → ; CDO → ; RER → ; VE → ; HRmax → ; d-ROMs → ; BAP →
						500 mL doses at 35 min before exercise		
Hori et al. (23)^b^	RCT	H:10	20.3 ± 1.3	Male:20(100) Female:0(0)	Untrained university students	HRW (H_2_ conc.:5.9 ppm) 500 mL on all weekdays for 2 weeks	Incremental cycling test to exhaustion	Fatigue: RPE → ; BLA^c^→ Aerobic Capacity: VO_2peak_→ Others: P_max_ → ; CDO → ; RER → ; VE → ; Resting HR → ; HRmax → ; d-ROMs↑; BAP↑
		P:10	20.4 ± 4.7					
Timon et al. (41)	RCD	27	25.5 ± 5.5 ^d^	Un	Trained cyclists (n=12) and untrained participants (n=15)	HRW (pH: 7.5; H_2_ conc.:1.9 ppm; ORP:−600 mV) 1920 and 2240 ml per day for 7 days	Incremental cycling test to exhaustion	Fatigue: RPE → ; BLA → Aerobic capacity: VO_2max_↑; TTE↑ Others: P_max_↑; HRmax → ; VT2 % VO_2max_↑; Fatigue index↓
			26.3 ± 5.9 ^e^					
Alharbi et al.(49)	RCD	18	21 ± 1	Male:18(100) Female:0(0)	Recreationally trained participants	HRC (0.636 μg/capsule) 2.544 μg/day for 3 days	Incremental cycling test to exhaustion	Fatigue: BLA → Aerobic capacity: VO_2peak_ → ; TTE → Others: Electrolytes (Na^+^ → ; K^+^ → ; Ca^2+^ → ; Cl^−^ → ; AGap↑; AGapK →); VE↑; VO_2_↑; VCO_2_↑; Blood gas (pH ↑; PO_2_ → ; PCO_2_ → ; HCO${}_{3}^{-}↑$); TR-NIRS in the RF/VL (Total [Hb+Mb] → ; Deoxy [Hb+Mb] ↑; StO_2_↑); HRmax → ; HR_avg_ → ; P_max_→
Dong et al. (46)	RCT	H:9	23.2 ± 1.1	Male:6(67) Female:3(33)	Dragon boat athletes	HRW (FH:1600 ppb) 1000 mL per day for 8 days	Rowing dynamometer rowing test	Aerobic capacity: Pm → Others: P_max_↑; HR_max_↓; Recovery HR ↓
s		P:9	22.7 ± 0.9	Male:6(67) Female:3(33)				
Botek et al. (24)	RCD	16	18.8 ± 1.2	Male:16(100) Female:0(0)	Professional soccer players	HRW (pH:7.9 ORP: −652 mV; Temp: 20°C; H_2_ conc.:0.9 ppm) 420 mL at 120 min, 60 min and 210 mL at 15 min, 5 min before exercise	Repeated sprints (15 × 30 m track sprints with recovery 20-s)	Fatigue: RPE → ; BLA → Aerobic capacity: Average time for 30m repeat sprints↑ Others:0~15m sprint time → ;15~30m sprint time →

a Study I in the publication; AGap, Aniongap; AGapK, Aniongap potassium;

b Study II in the publication; BLA, Blood lactate; BAP: Biological Antioxidant Potential; conc., concentration;

c Outcome data was not available by contacting the corresponding author and other authors on the publication; CK, Creatine kinase; CKa, Creatine kinase activity, CMJ, Countermovement Jump; CDO, Carbon Dioxide Output;

d cyclists; d-ROMs, diacron-Reactive Oxygen Metabolites;

e untrained participants;

ESR, Erythrocyte Sedimentation Rate; FH, Free Hydrogen; H, H_2_ group; HRW, Hydrogen-Rich Water; HRG, Hydrogen-Rich Gas; HRC, Hydrogen-Rich Calcium powder; HRG: Hydrogen-Rich Gas; HR, Heart Rate; HR_avg_, average Heart Rate; HR_max_, maximum Heart Rate; LDa, Lactate Dehydrogenase activity; MF, Median frequency; MPF, Mean Power Frequency; MVIS, Maximal Voluntary Isometric Strength; MRS, Maximal Running Speed; ORP, Oxidation Reduction Potential; OBLA, Onset of Blood Lactate Accumulation at 4 mmol·L-1;P, Placebo group; P_m_, mean Power; P_max_, maximum Power; RCD, Randomized crossover design; RCT, Randomized controlled trial; RE, Running Economy; rep., repetitions; RER, Respiratory Exchange Ratio; RR, Respiratory Rate; RQ, Respiratory Quotient; RPE, Rating of Perceived Exertion; RF, Rectus Femoris muscle; SCKa, Serum creatine kinas activity; SLDa, Serum lactate dehydrogenase activity; Temp, Temperature; TTE, Time-to-exhaustion; TDS, Total Dissolved Solids; TR-NIRS, Time-resolved Near-infrared Spectroscopy; U8ER, Urinary 8-hydroxydeoxyguanosine Excretion Rate; Un, Unreported; u, untrained; VO_2max_, Maximum Oxygen Uptake; VO_2peak_, Peak Oxygen Uptake; VO_2_, Oxygen Uptake; VT2 % VO_2max_, Percentage of maximal oxygen uptake in the ventilatory anaerobic threshold; VE, Ventilation volume; VAS, Visual Analog Scales; VL, Vastus Lateralis muscle; ↓, H_2_ significantly(p < 0.05) reduced the outcome compared to placebo; ↑, H_2_ significantly(p < 0.05) improved the outcome compared to placebo; → , no significant difference (p > 0.05) between H_2_ and placebo.

### 3.1. Participant characteristics

A total of 402 healthy participants with mean ages ranging from 18.8 to 51.5 years were included (Table 1). For the training status, 210 of them were untrained, and 192 were trained. Sex information was missing in one study (41) (Table 1).

### 3.2. Protocol of H_2_ administration

The included studies implemented three types of hydrogen, that is, hydrogen-rich water (HRW) (*n* = 13) (23–26, 28, 30, 41–47), hydrogen-rich gas (HRG) (*n* = 3) (27, 29, 48), and hydrogen-rich calcium powder (HRC) (*n* = 1) (49). Hydrogen concentrations varied considerably (e.g., HRW: 0.5~5.9 ppm; HRG: 1 to 68%) across these studies (Table 1). Eight studies (23, 25, 26, 28, 30, 42, 43, 50) examined the effects of a single intake of H_2_ within 24 h before exercise. Seven studies (23, 27, 41, 44–46, 49) implemented the protocol of repeatedly intaking H_2_ from 2 to 14 days before exercise. One study (47) used the intake of HRW before and after exercise for 3 days, and another study (48) used a single 60-min inhalation of HRG immediately after exercise. The specific amount of H_2_ was presented in Table 1. Placebos used in these studies depended upon the type of supplement and drinking water, inhaling normal air, or swallowing capsules (i.e., capsules containing calcium powder).

### 3.3. Exercise protocol

Two exercise types (continuous and intermittent) were used to induce fatigue. Specifically, eight studies (23, 25–28, 41, 45, 49) used continuous load-incremental exercise (i.e., incremental treadmill running test, incremental cycling test); four studies (29, 42, 46, 48) used continuous fixed-load exercise; one study (30) used a 4.2-km up-hill race; three studies (24, 44, 47) used intermittent sprint exercises; and the other one (43) used an intermittent judo fitness test.

### 3.4. Outcome measurements

Nine of the studies (23–25, 28, 30, 41, 43, 44, 49) assessed both fatigue and aerobic capacity immediately after exercise. Three studies (26, 42, 47) assessed only fatigue immediately after exercise. Four studies (29, 45, 46, 48) assessed only aerobic capacity. In addition, other outcomes such as heart rate, explosive power, respiratory circulations, blood metabolites, and muscle functions were also assessed (Table 1).

### 3.5. Risk of bias

**Figure 2:**
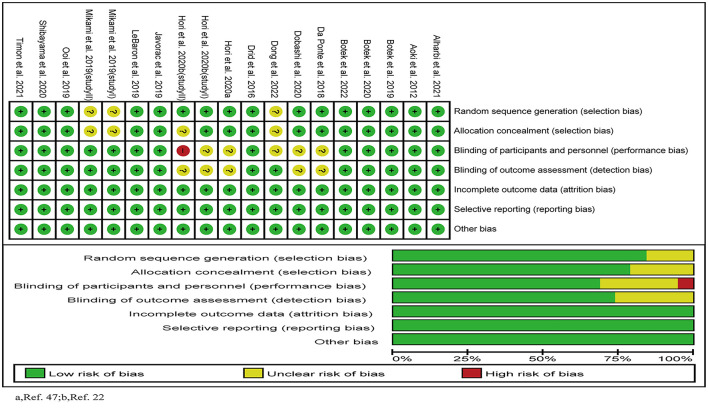
Risk of bias in the included studies.

The results of the quality evaluation of the included 19 studies were shown in Figure 2. Four (23, 29, 44, 47) of them used a randomized, single-blinded and placebo-controlled design; and the others used a randomized, double-blinded and placebo-controlled design. One study (23) was evaluated as high risk of bias, 11 were as low risk, and the other seven (23, 25, 29, 44, 46, 47) were as moderate risk.

### 3.6. Meta-analysis

Based on the heterogeneity, we performed subgroup analyses of RPE and blood lactate by comparing between training status (i.e., untrained and trained), intervention period (i.e., a single time within 24 h and multiple days before exercise), and exercise modes (i.e., continuous and intermittent exercises) as variables (Table 2).

**Table 2:** Subgroup analysis results regarding the effects of H_2_ on fatigue.

**Outcomes**	**Variables**	**No. of studies**	**SMD (95%CI)**	***P-*** **value**	**Test of heterogeneity**		
					χ^2^	**P-value**	**I** ^2^ **(%)**
RPE	**Training status**						
	Untrained	4	−0.47 (−0.78, −0.16)	0.003	1.70	0.637	0
	Trained	6	−0.36 (−0.75, 0.12)	0.161	11.40	0.044	56.1
	**Period of H**_**2**_ **implementation**						
	Intake H_2_ at a single time before exercise	7	−0.44 (−0.77, −0.12)	0.007	10.11	0.120	40.6
	Intake H_2_ for multiple days before exercise	2	−0.12 (−0.58, 0.33)	0.606	0	0.976	0
	**Exercise types**						
	Continuous exercises	8	−0.32 (−0.59, −0.06)	0.017	9.69	0.207	27.8
	Intermittent exercises	1	−0.96 (−1.70, −0.22)	0.011	0	–	–
Blood Lactate	**Training status**						
	Untrained	4	−0.30 (−0.69, 0.09)	0.132	0.77	0.856	0
	Trained	8	−0.49 (−0.92, −0.06)	0.025	14.73	0.040	52.5
	**Period of H**_**2**_ **implementation**						
	Intake H_2_ at a single time before exercise	6	−0.62 (−1.19, −0.05)	0.032	12.56	0.028	60.2
	Intake H_2_ for multiple days before exercise	5	−0.26 (−0.57, 0.06)	0.107	1.95	0.746	0
	**Exercise types**						
	Continuous exercises	7	−0.37 (−0.74, 0.00)	0.052	10.73	0.097	44.1
	Intermittent exercises	4	−0.56 (−1.12, 0.01)	0.053	4.37	0.224	31.3

#### 3.6.1. Effects of H_2_ on fatigue

##### 3.6.1.1. RPE score

Two publications (25, 26) showed that H_2_ can significantly reduce RPE score as compared to the placebo; while another six publications (23, 24, 28, 30, 41, 44) showed that H_2_ cannot significantly reduce RPE score (Table 1).

**Figure 3:**
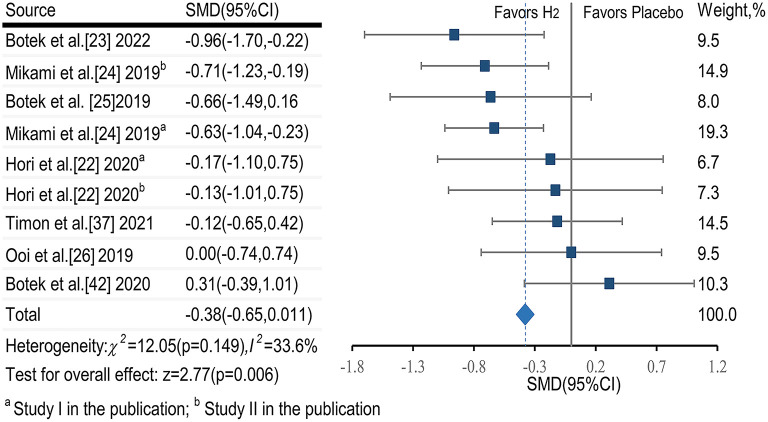
The pooled effect size of H_2_ intake on rating of perceived exertion.

The pooled ES of RPE score was small and significant (SMDpooled = −0.38, 95% CI −0.65 to −0.11, *p* = 0.006, Figure 3) with low heterogeneity (*I*^2^ = 33.6%, *p* = 0.149). The funnel plot (**Figure 7A**) and Egger's test (*t* = 0.98, *p* = 0.358) indicated no publication bias.

Subgroup analysis showed that participant training status contributed significantly to the effects of H_2_. Specifically, the ES in untrained participants was significant and small (SMD = −0.47, 95% CI −0.78 to −0.16, *p* = 0.003). The ES in trained participants was not significant and small (SMD = −0.36, 95% CI −0.75 to 0.12, *p* = 0.161). A significant and small ES for single-dose H_2_ intake before exercise (SMD = −0.44, 95% CI −0.77 to −0.12, *p* = 0.007) was observed, while only trivial ES was observed in multiple-day intakes of H_2_ (SMD = −0.12, 95% CI −0.58 to 0.33, *p* = 0.606). With respect to exercise type, the ES was large for intermittent exercises (SMD = −0.96, 95% CI −1.70 to −0.22, *p* = 0.011), and was small and significant (SMD = −0.32, 95% CI −0.59 to −0.06, *p* = 0.017) for continuous exercises.

##### 3.6.1.2. Blood lactate

Three publications (26, 42, 43) showed that H_2_ can significantly reduce blood lactate levels as compared to the placebo; while another eight publications (23, 24, 27, 28, 41, 44, 47, 49) showed opposite results that H_2_ cannot significantly reduce blood lactate levels (Table 1).

**Figure 4:**
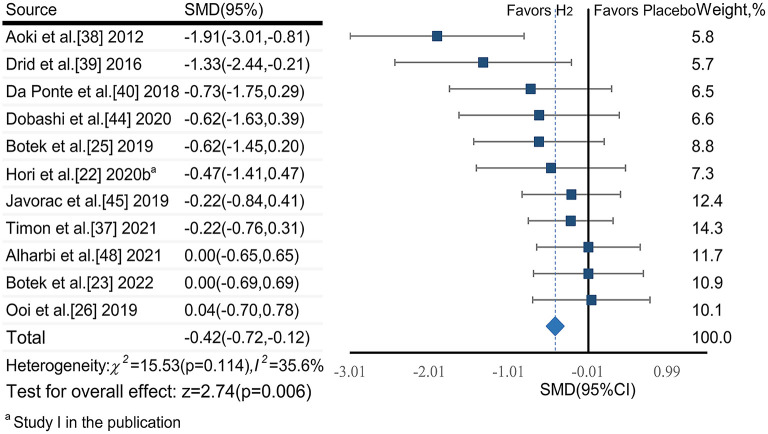
The pooled effect size of H_2_ intake on blood lactate.

The pooled ES of blood lactate was significant and small (SMD_pooled_ = −0.42, 95% CI −0.72 to −0.12, *p* = 0.006, Figure 4) with low heterogeneity (*I*^2^ = 35.6%, *p* = 0.114). The funnel plot (**Figure 7B**) and Egger's test (*t* = −3.64, *p* = 0.005) indicated a potential risk of publication bias; but the Trim and Fill method for sensitive analysis showed that the pooled ES (Fixed: SMD_pooled_ = –.362, *p* = 0.002; Random: SMD_pooled_ = −0.418, *p* = 0.006) was robust.

Subgroup analysis showed that participant training status contributed significantly to the effects of H_2_. Specifically, the ES in trained participants was significant and close to moderate (SMD = −0.49, 95% CI −0.92 to −0.06, *p* = 0.025), but was small and not significant in untrained participants (SMD = −0.30, 95% CI −0.69 to 0.09, *p* = 0.132). A significant and moderate ES for single-dose H_2_ intake before exercise (SMD = −0.62, 95% CI −1.19 to −0.05, *p* = 0.032) was observed, while only small ES was observed in multiple-day intakes of H_2_ (SMD = −0.26, 95% CI −0.57 to 0.06, *p* = 0.107). Regarding exercise type, the ES was moderate for intermittent exercises (SMD = −0.56, 95% CI −1.12 to 0.01, *p* = 0.053), and was small (SMD = −0.37, 95% CI −0.74 to 0.00, *p* = 0.052) for continuous exercises.

#### 3.6.2 Effects of H_2_ on aerobic capacity

##### 3.6.2.1. VO_2*max*_/VO_2*peak*_

Three publications (25, 29, 41) reported that H_2_ induced significant improvement in VO_2max_ or VO_2peak_ as compared to the placebo; while six publications (23, 25, 27, 28, 45, 49) reported no such effect (Table 1).

**Figure 5:**
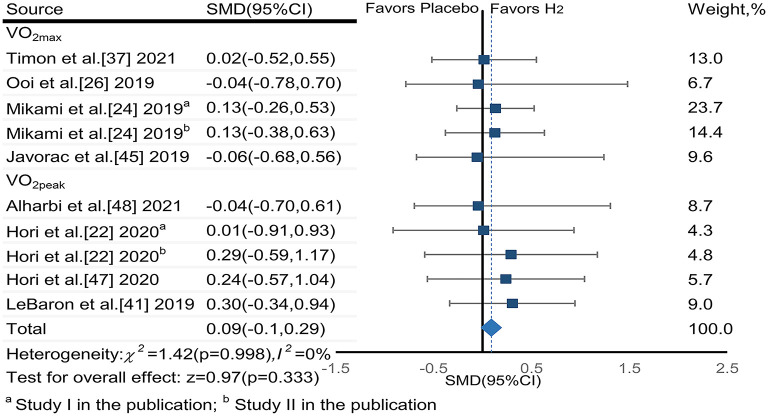
The pooled effect size of H_2_ intake on VO_2max_ (VO_2peak_).

The pooled ES of VO_2max_ and VO_2peak_ was not significant and trivial (SMD_pooled_ = 0.09,95% CI −0.10 to 0.29, *p* = 0.333, Figure 5) without heterogeneity (*I*^2^ = 0%, *p* = 0.996). The funnel plot (**Figure 7C**) and Egger's test (*t* = 0.01, *p* = 0.990) indicated no publication bias.

##### 3.6.2.2. Endurance performance

Two publications (24, 41) reported that H_2_ significantly increased cycling to exhaustion time or multiple repeat sprint performance as compared to the placebo; while another eight publications (27, 28, 30, 43, 44, 46, 48, 49) reported no such effect (Table 1).

**Figure 6:**
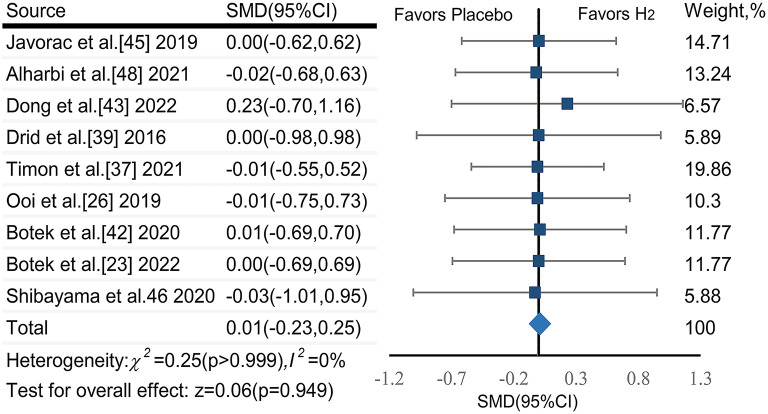
The pooled effect size of H_2_ intake on endurance performance.

**Figure 7:**
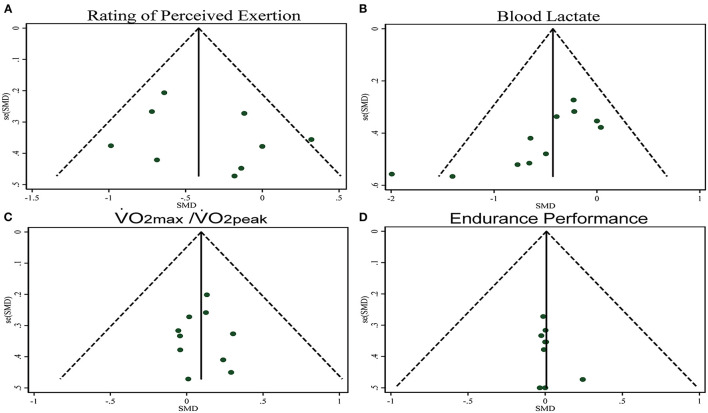
Funnel plots.

The pooled ES of endurance performance was not significant and trivial (SMD_pooled_ = 0.01,95% CI −0.23 to 0.25, *p* = 0.949, Figure 6) without heterogeneity (*I*^2^ = 0%, *p* > 0.999). The funnel plot (Figure 7D) and Egger's test (*t* = 1.18, *p* = 0.278) indicated no publication bias.

### 3.7. GRADE assessment

The quality of evidence was determined to be moderate, and details for the evaluation of the GRADE framework are presented in eTable 3.

## 4. Discussion

The results of this systematic review and meta-analysis suggest that H_2_ supplementation is a promising strategy for alleviating subjective fatigue and clearing blood lactate as induced by high-intensity exercise. However, H_2_ supplementation does not appear to enhance aerobic capacity. The quality of available evidence to date was moderate. Subgroup analyses revealed that the training status, the period of H_2_ implementation, and the type of exercise may all influence the effects of H_2_ on fatigue and thus need to be carefully considered in the design of future research and practice.

While our results indicate that H_2_ can significantly reduce subjective fatigue and blood lactate after high-intensity exercise in healthy adults, they do not provide evidence for the underlying bio-neurophysiological mechanisms involved. One possible explanation is that H_2_ appears to be a neuroprotective agent that facilitates the restoration of neuronal oxidative damage by reducing oxidative stress and neuroinflammation (51–54). H_2_ intake has also been reported to induce positive effects on exercise acidosis and reduce blood lactate concentration (26), thus modulating intracellular and extracellular buffering capacity during high-intensity exercise (55). It is also possible that the effects of H_2_ intake may depend upon the resting redox state of the user (56). Future studies are thus warranted to further investigate these potential pathways, which will ultimately help the design of appropriate strategies for fatigue alleviation using H_2_.

Subgroup analyses reveal several important factors that likely contribute to the effects of H_2_ supplementation on fatigue. First, we observed that the effects were greater in untrained as compared to trained individuals. This may be due to the antioxidant capacity during high-intensity exercise is relatively lower in untrained participants compared to trained participants (57), which may thus interfere with the effects of H_2_ on fatigue (30), presenting relatively smaller effect size of H_2_ on fatigue in this cohort (42, 47). Second, longer period of H_2_ implementation, or multiple intakes of H_2_, was not associated with greater reduction of fatigue in healthy adults, as compared to the intake of a single dose immediately before exercise. This observation may be due to the low between-day retention rate of H_2_; for example, it was observed that over 59% of H_2_ can be exhaled within the first hour after the intake of HRW (25). Third, subgroup analyzes revealed that H_2_ supplementation may be more effective for fatigue induced by intermittent exercise as compared to continuous exercise (24, 28, 30). This may be because bouts of intermittent exercise are typically completed against greater external physical load, which may enable mitochondrial respiratory function to more efficiently intake H_2_ and/or increase the concentration level of ROS in muscles, boosting the redox procedure between ROS and molecular hydrogen (58–60).

Intriguingly, H_2_ supplementation did not appear to significantly improve aerobic capacity. This suggests that the observed impact of H_2_ intake on fatigue during high-intensity exercise was not sufficient to translate into improved aerobic capacity in healthy adults. Aerobic capacity depends upon multiple underlying bio-physiological procedures, including respiratory function, regulation of oxygen, and local muscle oxygen utilization (61, 62). Previous studies have reported that acute H_2_ supplementation does not substantially modulate these critical factors of aerobic capacity (23, 26–28, 49), which may at least in part underlie its lack of effect on this important function in humans.

### 4.1. Limitations

Several of the included studies were conducted on a small number of participants (*n* ≤ 10) (23, 43, 44, 47, 48), which may lead to potential bias. Most included studies focused on only younger and middle-aged men. As such, future studies are thus needed to examine whether the effects of H_2_ supplementation differs by age and/or sex. With respect to the latter, studies have reported that compared to men, the antioxidant protective function in women is greater due to estrogen (63, 64), which may potentially influence the effects of H_2_ on fatigue. Finally, considerable work is still needed to determine the dose-response relationship between H_2_ and fatigue and the impact of such supplementation on physiological adaptation to exercise and the risk of injury over time.

## 5. Conclusions

This analysis indicates that H_2_ supplementation can alleviate fatigue but cannot significantly enhance aerobic capacity in healthy adults. The knowledge obtained from this study, such as the appropriate protocols of H_2_ administration and selection of exercise type to induce fatigue, will ultimately help inform future studies to confirm and explicitly examine the benefits of H_2_ on athletes and untrained people with more rigorous design (e.g., matched number of men and women), helping optimize the protocols of fatigue recovery in the daily routines of professional athletes and untrained people.

## Data availability statement

The original contributions presented in the study are included in the article/Supplementary material, further inquiries can be directed to the corresponding authors.

## Author contributions

KZ, ML, and DB had full access to all the data in the study and take responsibility for the integrity of the data and the accuracy of the data analysis. Concept and design: DB, KZ, and JZ. Acquisition, analysis, or interpretation of data: KZ, ML, YW, HL, and JZ. Drafting of the manuscript: KZ and ML. Critical revision of the manuscript for important intellectual content: KZ, ML, YW, HL, BM, DB, LZ, and JZ. Statistical analysis: YW, LZ, and HL. All authors contributed to the article and approved the submitted version.
