# Supplementary material for: Effects of molecular hydrogen supplementation on fatigue and aerobic capacity in healthy adults: A systematic review and meta-analysis
Source: Front Nutr. 2023 Feb 2;10:1094767. doi: 10.3389/fnut.2023.1094767 (PMC9934906; doi:10.3389/fnut.2023.1094767)
Supplement: Supplementary file 1 [file Table_1.DOCX]

**eTable 1 Search Strategy**

**eTable 2** **The post-test data of outcomes in each study**

**eTable 3 The quality of the evidence (GRADE)**

**eReferences**

**eTable 1 Search Strategy**

| Process | Keywords |
| --- | --- |
| # 1 | "molecular hydrogen" OR "hydrogen rich water" OR "hydrogen-rich water" OR "hydrogen rich saline" OR "hydrogen-rich saline" OR "H_2_-dissolved water" OR "H_2_-infused water" OR "hydrogen gas" OR "hydrogen inhalation" OR "hydrogen bathing" |
| # 2 | fatigue OR "fatigue perception" OR "Borg’s scale" OR "rating of perceived exertion" OR "visual analogue scale" OR "blood lactate" OR "blood lactic acid" |
| # 3 | "physical performance" OR "physical fitness testing" OR "physical exercise" OR "exercise capacity" OR "aerobic fitness" OR "aerobic performance" OR "aerobic capacity" OR "maximal oxygen uptake" OR "running economy" OR "running to exhaustion" OR "cycle ergometer" OR "bicycle ergometry test" OR "incremental cycling exercise test" OR "incremental treadmill test" OR "step tests" OR "field testing" OR "intermittent exercise" OR "repeated sprints" OR "swimming" |
| # 4 | # 2 OR # 3 |
| # 5 | # 1 AND # 4 |

**eTable 2 The post-test data of outcomes in each study**

| Study | Outcome | Mean (SD) of H_2_ group | Mean (SD) of placebo group |
| --- | --- | --- | --- |
| Mikami et al.[1] 2019^b^ | RPE ^c^ | 11.3 (1.2) | 12.2 (1.3) |
| Botek et al.[2] 2019 | RPE | 17.8 (1.2) | 18.5 (0.8) |
| Mikami et al.[1] 2019^a^ | RPE ^c^ | 12.5 (1.7) | 13.5 (1.4) |
| Hori et al.[3] 2020^a^ | RPE | 18.8 (1.8) | 19.1 (1.5) |
| Hori et al.[3] 2020^b^ | RPE | 18.8 (1.6) | 19.0 (1.3) |
| Timon et al.[4] 2021 | RPE | 8.2 (0.9) | 8.3 (0.8) |
| Ooi et al.[5] 2019 | RPE | 19.0 (1.0) | 19.0 (1.0) |
| Botek et al.[6] 2020 | RPE | 17.8 (1.2) | 17.4 (1.3) |
| Botek et al.[7] 2022 | RPE | 5.5 (0.7) | 6.1 (0.5) |
| Aoki et al.[8] 2012 | Blood Lactate(mmol/L) | 4.0 (0.6) | 5.3 (0.7) |
| Drid et al.[9] 2016 | Blood Lactate(mmol/L) | 6.8 (2.3) | 9.7 (1.8) |
| Da Ponte et al.[10] 2018 | Blood Lactate(mmol/L) | 14.5 (2.8) | 16.7 (2.9) |
| Dobashi et al.[11] 2020 | Blood Lactate(mmol/L) | 14.5 (2.0) | 16.1 (2.8) |
| Botek et al.[2] 2019 | Blood Lactate(mmol/L) | 8.9 (2.2) | 10.6 (3.0) |
| Hori et al.[3] 2020^a^ | Blood Lactate(mmol/L) | 10.8 (0.7) | 11.2 (0.9) |
| Javorac et al.[12] 2019 | Blood Lactate(mmol/L) | 1.8 (0.4) | 1.9 (0.5) |
| Timon et al.[4] 2021 | Blood Lactate(mmol/L) | 11.8 (3.9) | 12.6 (3.1) |
| Alharbi et al.[13] 2021 | Blood Lactate(mmol/L) | 5.6 (2.7) | 5.6 (3.1) |
| Botek et al.[7] 2022 | Blood Lactate(mmol/L) | 10.4 (3.2) | 10.4 (2.5) |
| Ooi et al.[5] 2019 | Blood Lactate(mmol/L) | 16.6 (2.7) | 16.5 (2.6) |
| Timon et al.[4] 2021 | VO_2max_(ml/min/kg) | 50.6 (11.8) | 50.4 (12.3) |
| Ooi et al.[5] 2019 | VO_2max_(ml/min/kg) | 56.9 (4.4) | 57.1 (4.7) |
| Mikami et al.[1] 2019^a^ | VO_2max_(ml/min/kg) ^c^ | 31.3 (3.8) | 30.8 (3.7) |
| Mikami et al.[1] 2019^b^ | VO_2max_(ml/min/kg) ^c^ | 39.3 (4.8) | 38.6 (6.1) |
| Alharbi et al.[13] 2021 | VO_2peak_(mL/min) | 3119.0 (423.0) | 3141.0 (546.0) |
| Javorac et al.[12] 2019 | VO_2max_(ml/min/kg) ^c^ | 39.2 (7.1) | 39.6 (7.1) |
| Hori et al.[3] 2020^a^ | VO_2peak_(mL/min) | 2819.0 (585.0) | 2813.0 (549.0) |
| Hori et al.[3] 2020^b^ | VO_2peak_(mL/min) | 3202.0 (246.7) | 3095.0 (433.2) |
| LeBaron et al.[14] 2019 | VO_2peak_(mL/min/kg) | 38.1 (5.6) | 36.3 (6.0) |
| Hori et al.[15] 2020 | VO_2peak_(mL/min) | 2674.4 (563.9) | 2534.9 (563.9) |
| Javorac et al.[12] 2019 | Time to exhaustion (min) ^c^ | 401.4 (86.6) | 401.2 (86.7) |
| Alharbi et al.[13] 2021 | Time to exhaustion (min) | 26.8 (4.0) | 26.9 (3.9) |
| Dong et al.[16] 2022 | Average power of rowing (watt) | 321.3 (77.5) | 296.2 (123.5) |
| Drid et al.[9] 2016 | Fitness performance index | 13.4 (1.0) | 13.4 (1.0) |
| Timon et al.[4] 2021 | Cycling time to exhaustion(s) | 728.8 (248.4) | 731.9 (242.8) |
| Ooi et al.[5] 2019 | Running time to exhaustion(s) | 618.0 (126.0) | 619.0 (113.0) |
| Botek et al.[6] 2020 | 4.2-km up-hill races time (s) | 1249.0 (163.0) | 1250.0 (173.0) |
| Botek et al.[7] 2022 | 15*30 m sprint average time(s) | 4.5 (0.1) | 4.5 (0.1) |
| Shibayama et al.[17] 2020 | Average power of cycling (watt) | 573.4 (76.1) | 576.1 (80.0) |

Data extraction instructions: The mean and standard deviation of each outcome in post-tests were extracted for each study. If these values were unavailable, they were calculated using the following formulas, where the correlation coefficient (Corr) was set at 0.5 [18, 19]. When any relevant data was missing, we tried to request it by contacting the corresponding author and other authors on the publication[18].

$Meanpost=Meanpre+Meanchang$;$\mathrm{SDpost}=\frac{2\times\mathrm{Corr}\times\mathrm{SDpre}+\sqrt{4\times\mathrm{Corr}^{2}\times\mathrm{SDpre}^{2}-4\times(\mathrm{SDpre}^{2}-SDchange^{2})}}{2}$

^a^, Study I in the publication; ^b^, Study II in the publication; SD, Standard deviation; ^c^, Post-test data for the outcome was calculated from the above equations.

**eTable 3 The quality of the evidence (GRADE)**

| **Quality assessment** | | | | | | | **No of participants** | | **Effect** | **Quality** | **Importance** |
| --- | --- | --- | --- | --- | --- | --- | --- | --- | --- | --- | --- |
| **No of experiments** | **Design** | **Risk of bias** | **Inconsistency** | **Indirectness** | **Imprecision** | **Other considerations** | **H2** | **Placebo** | **Absolute** |  |  |
| **RPE (Better indicated by lower values)** | | | | | | | | | | | |
| 9 | randomised trials | serious^1,2^ | no serious inconsistency | no serious indirectness | no serious imprecision | none | 186 | 181 | SMD 0.38 lower (0.65 to 0.11 lower) | ⊕⊕⊕⊝ | CRITICAL |
|  |  |  |  |  |  |  |  |  |  | MODERATE |  |
| **Blood Lactate (Better indicated by lower values)** | | | | | | | | | | | |
| 11 | randomised trials | no serious risk of bias^1,2^ | no serious inconsistency | no serious indirectness | no serious imprecision | reporting bias^3^ | 150 | 150 | SMD 0.42 lower (0.72 to 0.12 lower) | ⊕⊕⊕⊝ | IMPORTANT |
|  |  |  |  |  |  |  |  |  |  | MODERATE |  |
| **VO2max/VO2peak (Better indicated by lower values)** | | | | | | | | | | | |
| 9 | randomised trials | serious^1,2^ | no serious inconsistency | no serious indirectness | no serious imprecision | none | 211 | 206 | SMD 0.09 higher (0.10 lower to 0.29 higher) | ⊕⊕⊕⊝ | CRITICAL |
|  |  |  |  |  |  |  |  |  |  | MODERATE |  |
| **Aerobic Performance (Better indicated by lower values)** | | | | | | | | | | | |
| 9 | randomised trials | serious^1,2,3^ | no serious inconsistency | no serious indirectness | no serious imprecision | none | 136 | 136 | SMD 0.01 higher (0.23 lower to 0.25 higher) | ⊕⊕⊕⊝ | IMPORTANT |
|  |  |  |  |  |  |  |  |  |  | MODERATE |  |

^1^ Risk of performance bias
^2^ Risk of selective bias
^3^ No explanation was provid

**eReferences**

1. Mikami T, Tano K, Lee H, Lee H, Ohta SJCJoP, Pharmacology. Drinking Hydrogen Water Enhances Endurance and Relieves Psychometric Fatigue: Randomized, Double-blind, Placebo-controlled Study. 2019;97(9).

2. Botek M, Krejčí J, McKune AJ, Sládečková B, Naumovski N. Hydrogen Rich Water Improved Ventilatory, Perceptual and Lactate Responses to Exercise. Int J Sports Med. 2019;40(14):879-85.<https://doi.org/10.1055/a-0991-0268>

3. Hori A, Sobue S, Kurokawa R, Hirano SI, Ichihara M, Hotta N. Two-week continuous supplementation of hydrogenrich water increases peak oxygen uptake during an incremental cycling exercise test in healthy humans: a randomized, single-blinded, placebo-controlled study. Med Gas Res. 2020;10(4):163-9.<https://doi.org/10.4103/2045-9912.304223>

4. Timon R, Olcina G, Gonzalez-Custodio A, Camacho-Cardenosa M, Camacho-Cardenosa A, Martinez Guardado I. Effects of 7-day intake of hydrogen-rich water on physical performance of trained and untrained subjects. Biol Sport. 2021;38(2):269-75.<https://doi.org/10.5114/biolsport.2021.99705>

5. Ooi CH, Ng SK, Omar EAJAPN, Metabolism. Acute ingestion of hydrogen-rich water does not improve incremental treadmill running performance in endurance-trained athletes. 2019;45(5).

6. Botek M, Krejčí J, McKune AJ, Sládečková B. Hydrogen-Rich Water Supplementation and Up-Hill Running Performance: Effect of Athlete Performance Level. Int J Sports Physiol Perform. 2020:1-4.<https://doi.org/10.1123/ijspp.2019-0507>

7. Botek M, Khanna D, Krejčí J, Valenta M, McKune A, Sládečková B, et al. Molecular Hydrogen Mitigates Performance Decrement during Repeated Sprints in Professional Soccer Players. Nutrients. 2022;14(3).<https://doi.org/10.3390/nu14030508>

8. Aoki K, Nakao A, Adachi T, Matsui Y, Miyakawa S. Pilot study: Effects of drinking hydrogen-rich water on muscle fatigue caused by acute exercise in elite athletes. Med Gas Res. 2012;2:12.<https://doi.org/10.1186/2045-9912-2-12>

9. Drid P, Trivic T, Casals C, Trivic S, Stojanovic M, Ostojic SM. Is molecular hydrogen beneficial to enhance post-exercise recovery in female athletes? Sci Sports. 2016;31(4):207-13.<https://doi.org/10.1016/j.scispo.2016.04.010>

10. Da Ponte A, Giovanelli N, Nigris D, Lazzer S. Effects of hydrogen rich water on prolonged intermittent exercise. J Sports Med Phys Fitness. 2018;58(5):612-21.<https://doi.org/10.23736/s0022-4707.17.06883-9>

11. Dobashi S, Takeuchi K, Koyama K. Hydrogen-rich water suppresses the reduction in blood total antioxidant capacity induced by 3 consecutive days of severe exercise in physically active males. Med Gas Res. 2020;10(1):21-6.<https://doi.org/10.4103/2045-9912.279979>

12. Javorac D, Stajer V, Ratgeber L, Betlehem J, Ostojic S. Short-term H(2) inhalation improves running performance and torso strength in healthy adults. Biol Sport. 2019;36(4):333-9.<https://doi.org/10.5114/biolsport.2019.88756>

13. Alharbi AAD, Ebine N, Nakae S, Hojo T, Fukuoka Y. Application of Molecular Hydrogen as an Antioxidant in Responses to Ventilatory and Ergogenic Adjustments during Incremental Exercise in Humans. Nutrients. 2021;13(2).<https://doi.org/10.3390/nu13020459>

14. LeBaron TW, Larson AJ, Ohta S, Mikami T, Barlow J, Bulloch J, et al. Acute Supplementation with Molecular Hydrogen Benefits Submaximal Exercise Indices. Randomized, Double-Blinded, Placebo-Controlled Crossover Pilot Study. J Lifestyle Med. 2019;9(1):36-43.<https://doi.org/10.15280/jlm.2019.9.1.36>

15. Hori A, Ichihara M, Kimura H, Ogata H, Kondo T, Hotta N. Inhalation of molecular hydrogen increases breath acetone excretion during submaximal exercise: a randomized, single-blinded, placebo-controlled study. Med Gas Res. 2020;10(3):96-102.<https://doi.org/10.4103/2045-9912.296038>

16. Dong G, Fu J, Bao D, Zhou J. Short-Term Consumption of Hydrogen-Rich Water Enhances Power Performance and Heart Rate Recovery in Dragon Boat Athletes: Evidence from a Pilot Study. Int J Env Res Public Health. 2022;19(9).<https://doi.org/10.3390/ijerph19095413>

17. Shibayama Y, Dobashi S, Arisawa T, Fukuoka T, Koyama K. Impact of hydrogen-rich gas mixture inhalation through nasal cannula during post-exercise recovery period on subsequent oxidative stress, muscle damage, and exercise performances in men. Med Gas Res. 2020;10(4):155-62.<https://doi.org/10.4103/2045-9912.304222>

18. Higgins JP, Thomas, J., Chandler, J., Cumpston, M., Li, T., Page, M.J., et al. Cochrane handbook for systematic reviews of interventions. 2rd ed. UK: John Wiley & Sons; 2019.

19. Xue Y, Yang Y, Huang T. Effects of chronic exercise interventions on executive function among children and adolescents: a systematic review with meta-analysis. Br J Sports Med. 2019;53(22):1397-404.<https://doi.org/10.1136/bjsports-2018-099825>
